# Crystal structure of a layered coordination polymer based on a 4^4^ net containing Cd^2+^ ions and 1,5-bis­(pyridin-4-yl)pentane linkers

**DOI:** 10.1107/S1600536814014779

**Published:** 2014-07-19

**Authors:** William T. A. Harrison, M. John Plater, Ben M. deSilva deSilva, Mark R. St J. Foreman

**Affiliations:** a Department of Chemistry, University of Aberdeen, Meston Walk, Aberdeen AB24 3UE, Scotland

**Keywords:** Cadmium, flexible ligand, layered coordination polymer, crystal structure

## Abstract

The title compound, poly[[di­aqua­bis­[1,5-bis­(pyridin-4-yl)pentane-κ^2^ *N*:*N*′]cadmium] bis­(perchlorate) 1,5-bis­(pyridin-4-yl)pentane ethanol mono­solvate], [Cd(C_15_H_18_N_2_)_2_(H_2_O)_2_](ClO_4_)_2_·C_15_H_18_N_2_·C_2_H_6_O, is a layered coordination polymer built up from highly squashed 4^4^ nets in which the octa­hedral *trans*-CdO_2_N_4_ nodes (Cd site symmetry -1) are linked by the bifunctional ligands, forming infinite (110) sheets. The cationic sheets are charge-balanced by inter­layer perchlorate ions. A free 1,5-bis­(pyridin-4-yl)pentane mol­ecule and an ethanol mol­ecule of crystallization are also found in the inter­sheet region. A number of O—H⋯O, O—H⋯N and C—H⋯O hydrogen bonds help to consolidate the layered structure.

## Abstract 2

[Cd(C_15_H_18_N_2_)_2_(H_2_O)_2_](ClO_4_)_2_·C_15_H_18_N_2_·C_2_H_6_O, is a layered coordination polymer containing highly squashed 4^4^ nets. The polymeric sheets alternate with layers of counter ions, free ligands and solvent molecules.

## Chemical context   

The most popular linking ligands in metal-organic frameworks (MOFs) are probably multi-functional carboxyl­ates (Batten *et al.*, 2009 ▶) but other functional groups are also possible. As part of our ongoing studies of flexible bifunctional pyridyl ligands (Plater *et al.*, 2008 ▶) as potential MOF linkers, we now describe the synthesis and structure of the title layered coordination polymer, (I), which combines Cd^2+^ ions and the little-studied ligand 1,5-bis­(pyridin-4-yl)pentane, C_15_H_18_N_2_. The neutral bridging ligand necessitates the presence of perchlorate counter-ions (from the starting metal salt), which exert an important influence on the structure.
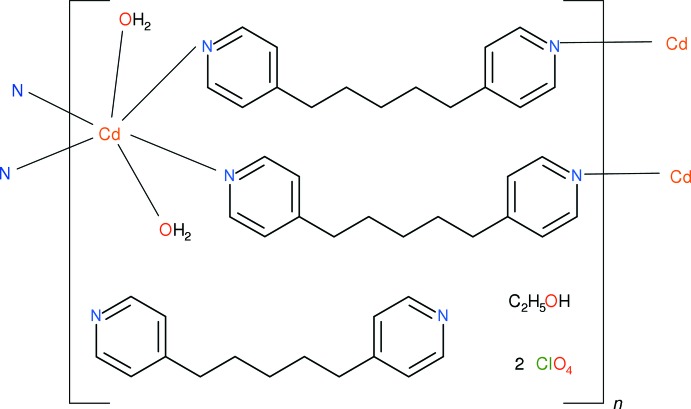


## Structural commentary   

**Figure 1:**
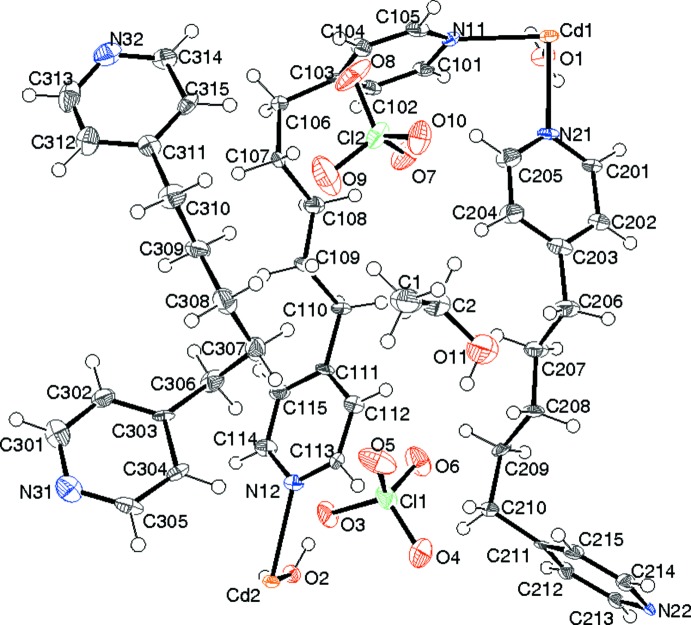
The asymmetric unit of (I) showing 50% displacement ellipsoids.

The asymmetric unit of (I) contains two Cd^2+^ ions (both lying on crystallographic inversion centres), three 1,5-bis­(pyridin-4-yl)pentane (C_15_H_18_N_2_; *L*) molecules, two perchlorate ions, two water mol­ecules and one ethanol mol­ecule (Fig. 1 ▶). The cadmium ions, water mol­ecules and two of the *L* molecules combine to generate an infinite cationic network of compos­ition [Cd(H_2_O)_2_ *L*_2_]^2+^_*n*_.

**Figure 2:**
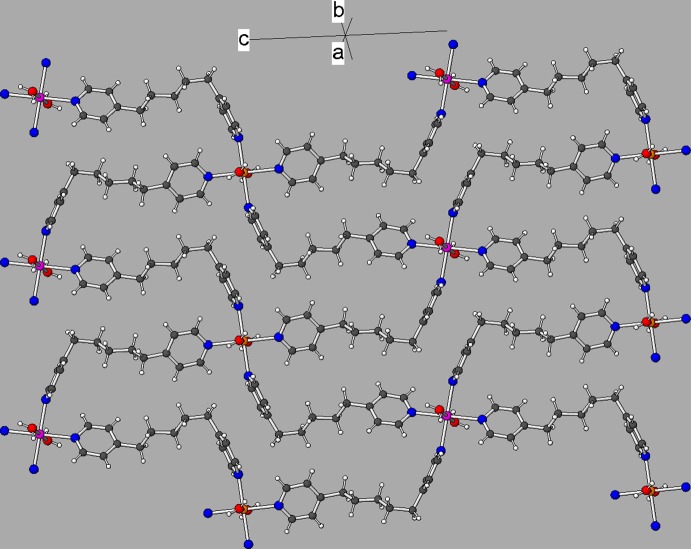
Part of an infinite 4^4^ sheet propagating in (110) in the structure of (I). The Cd1 and Cd2 ions are represented by orange and fuchsia spheres, respectively.

Both cadmium ions adopt almost regular *trans*-CdO_2_N_4_ octa­hedral coordination geometries (Table 1 ▶) arising from two water mol­ecules and four ligands. The mean Cd—O and Cd—N bond lengths are 2.327 and 2.341 Å, respectively. Bond-valence sum (BVS) calculations (Brese & O’Keeffe, 1991 ▶) in valence units for Cd1 and Cd2 yield values of 2.11 and 2.02, respectively, in close agreement with the expected value of 2.00. The octa­hedral angular variances (Robinson *et al.*, 1971 ▶) for Cd1 and Cd2 are 2.53 and 10.57°^2^, respectively. Both ligands bridge the Cd1 and Cd2 atoms, resulting in a highly squashed and contorted 4^4^ network (O’Keeffe & Hyde, 1996 ▶), which propagates in the (110) plane, as shown in Fig. 2 ▶: each Cd1 atom is linked to four different Cd2 atoms and *vice versa*. The shortest Cd1⋯Cd2 separations (*via* ligands) are 14.4350 (6) and 14.7807 (6) Å. The shortest non-bonded Cd1⋯Cd1 and Cd2⋯Cd2 separations across a squashed 4^4^ square are both 11.0921 (5) Å. It is inter­esting that the shortest metal–metal distances in (I) of 10.0618 (4) and 10.1653 (4) Å for both Cd1 and Cd2 are inter-sheet separations.

For the N11 ligand mol­ecule, the dihedral angle between the N11 and N12 rings is 77.8 (4)° and the alkyl chain adopts a *gaaa* (*g* = *gauche*, *a* = *anti*) conformation (reading from the N11 ring to the N12 ring). Cd1 is displaced by 0.69 (1) Å from the N11 ring plane and Cd2 is displaced by −0.26 (1) Å from the N12 plane. In the N21 ligand mol­ecule, the dihedral angle between the pyridine rings is 75.2 (4)° and the alkyl-chain conformation is *aaag* (in the sense of the N21 ring to the N22 ring). The displacement of Cd1 from the N21 ring is 0.42 (1) Å and the displacement of Cd2 from the N22 ring is −0.58 (1) Å. The shortest out-and-back pathway from any metal atom to itself encompasses no fewer than 56 atoms (4 metal atoms and 4 × 13 ligand atoms).

**Figure 3:**
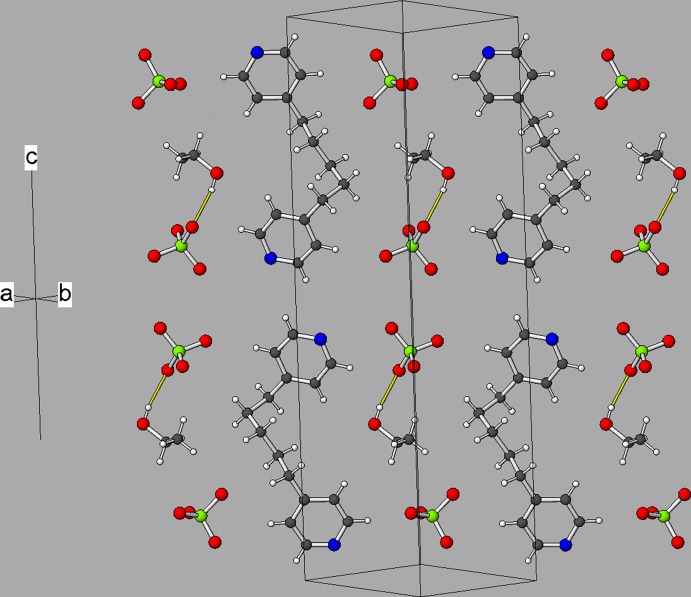
Part of a layer of perchlorate ions, N31-ligands and ethanol mol­ecules in the structure of (I). The O_e_—H⋯O (e = ethanol) hydrogen bond is shown as a yellow line.

The mean Cl—O bond lengths in the perchlorate ions in (I) are 1.446 Å for the Cl1 species and 1.436 Å for the Cl2 species. The third (N31) ligand mol­ecule is not bonded to the metal ions: the dihedral angle between its N31 and N32 rings is 18.3 (5)° and its alkyl chain conformation is *ggaa* (from N31 to N32; Fig. 3 ▶).

## Supra­molecular features   

**Figure 4:**
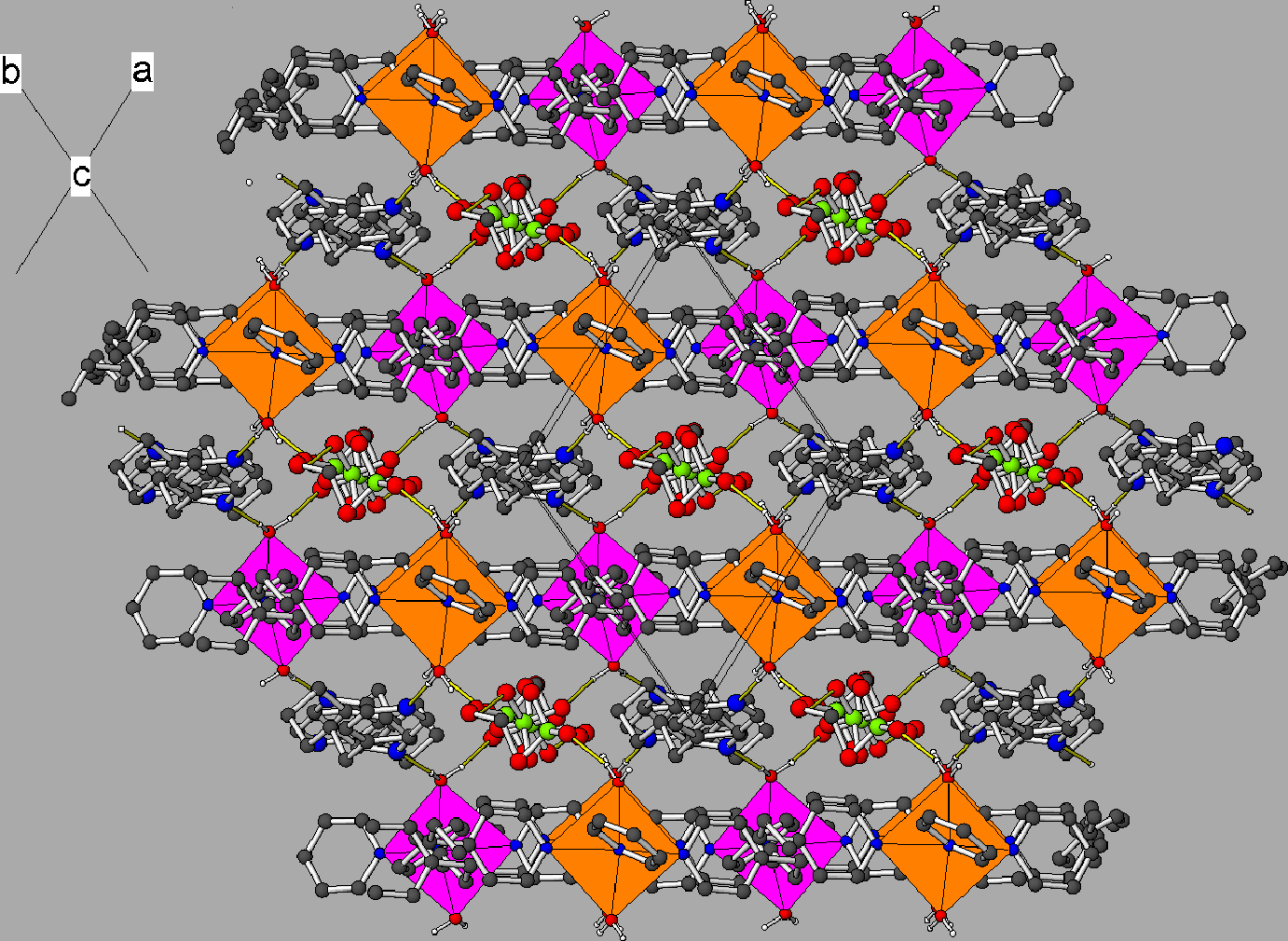
View down [001] of the structure of (I) showing the alternating polymeric [Cd(H_2_O)_2_ *L*_2_]_*n*_ and perchlorate/solvent molecule layers. The Cd1- and Cd2-centred octa­hedra are shown as orange and fuchsia polyhedra, respectively.

In the crystal, the infinite [Cd(H_2_O)_2_ *L*_2_]_*n*_ sheets propagate in the (110) plane (Fig. 4 ▶). There is no inter­penetration of the sheets in this structure. Sandwiched between the cationic sheets are layers of perchlorate ions, free (unbounded) N31-molecules and ethanol solvent mol­ecules. The water mol­ecules attached to the cadmium ions each form one O—H⋯O hydrogen bond to a perchlorate ion and one O—H⋯N hydrogen bond to the free solvent mol­ecule, such that both N31 and N32 accept a hydrogen bond. An intra-layer O_e_—H⋯Cl (e = ethanol) hydrogen bond also occurs. A number of C—H⋯O inter­actions are also observed (mean H⋯O = 2.54 Å): see Table 2 ▶.

## Database survey   

Only four ‘hits’ for crystal structures containing 1,5-bis(pyridin-4-yl)pentane were obtained from a search of Version 5.31 (last update February 2014) of the Cambridge Structural Database (Allen & Motherwell, 2002 ▶). Three of these are the isostructural family [*M*(C_15_H_18_N_2_)_2_(NO_3_)_2_]_*n*_, (*M* = Co, Ni, Cu) (Plater *et al.*, 2008 ▶), which contain inter­penetrated 6^5^.8 nets, with the nitrate counter-ions directly bonded to the metal ions. In [Cd_4_(C_15_H_18_N_2_)_8_(NO_3_)_8_]_*n*_·2*n*H_2_O, (II), (Plater *et al.*, 2000 ▶), remarkable triply-inter­penetrated 6^3^ nets occur in which the cadmium ions are coordinated by three ligand N atoms and two *O*,*O*-bidentate nitrate ions, generating distorted CdN_3_O_4_ penta­gonal bipyramids. It may be noted that in (I) and (II) the counter-ions and water mol­ecules have effectively swapped places, resulting in radically different structures.

## Synthesis and crystallization   

1,5-Bis(pyridin-4-yl)pentane (0.1 g, 0. 450 mmol; Plater *et al.*, 2000 ▶) was dissolved in ethanol (5 ml) and carefully layered onto a solution of Cd(ClO_4_)_2_·*x*H_2_O (0.137 g, 0.44 mmol) in water (5 ml). The solution was left to stand for two weeks during which time colourless blocks of (I) grew at the layer inter­face. The crystals were harvested and air dried (0.107 g, 45%). IR (KBr disc)/cm^−1^ ν = 3469 *s*, 3422 *s*, 2932 *s*, 2858 *s*, 1513 *s*, 1427 *s*, 1226 *s*, 1094 *s*, 1012 *w*, 842 *w*, 800 *w*, 624 *s* and 512 *w*.

## Refinement   

The O-bound H atoms were located in difference maps and refined as riding atoms in their as-found relative positions. The C-bound H atoms were placed geometrically and refined as riding atoms. The H atoms of the methyl group were allowed to rotate, but not to tip, to best fit the electron density. The constraint *U*_iso_(H) = 1.2*U*_eq_(C,O) or 1.5*U*_eq_(methyl C) was applied in all cases. Crystal data, data collection and structure refinement details are summarized in Table 3 ▶.

## Supplementary Material

Crystal structure: contains datablock(s) I. DOI: 10.1107/S1600536814014779/wm0007sup1.cif

Structure factors: contains datablock(s) I. DOI: 10.1107/S1600536814014779/wm0007Isup2.hkl

CCDC reference: 1008978

Additional supporting information:  crystallographic information; 3D view; checkCIF report

## Figures and Tables

**Table 1:** Selected bond lengths (Å)

Cd1—O1	2.317 (5)	Cd2—O2	2.337 (5)
Cd1—N11	2.319 (7)	Cd2—N22^i^	2.333 (6)
Cd1—N21	2.349 (6)	Cd2—N12	2.363 (6)

Symmetry code: (i) 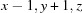.

**Table 2:** Hydrogen-bond geometry (Å, °)

*D*—H⋯*A*	*D*—H	H⋯*A*	*D*⋯*A*	*D*—H⋯*A*
O1—H1*O*⋯O8^ii^	0.86	1.96	2.795 (9)	165
O1—H2*O*⋯N32^iii^	0.84	1.87	2.705 (10)	175
O2—H3*O*⋯N31^ii^	0.86	1.91	2.736 (9)	162
O2—H4*O*⋯O3^iv^	0.84	2.20	2.880 (8)	138
O11—H11⋯O6	0.84	2.14	2.910 (11)	152
C1—H1*C*⋯O5	0.98	2.57	3.493 (12)	157
C101—H101⋯O1	0.95	2.55	3.226 (10)	128
C113—H113⋯O6	0.95	2.56	3.257 (12)	130
C201—H201⋯O10^ii^	0.95	2.54	3.260 (11)	133
C205—H205⋯O7	0.95	2.55	3.214 (12)	127
C214—H214⋯O2^v^	0.95	2.52	3.201 (11)	128
C304—H304⋯O3	0.95	2.47	3.420 (11)	174

Symmetry codes: (ii) 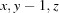; (iii) 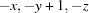; (iv) 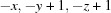; (v) 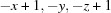.

**Table 3:** Experimental details

Crystal data	
Chemical formula	[Cd(C_15_H_18_N_2_)_2_(H_2_O)_2_](ClO_4_)_2_·C_15_H_18_N_2_·C_2_H_6_O
*M*_r_	1072.34
Crystal system, space group	Triclinic, *P* 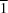
Temperature (K)	120
*a*, *b*, *c* (Å)	10.0618 (3), 10.1653 (3), 27.0304 (11)
α, β, γ (°)	87.163 (1), 85.001 (1), 66.509 (1)
*V* (Å^3^)	2525.60 (15)
*Z*	2
Radiation type	Mo *K*α
μ (mm^−1^)	0.60
Crystal size (mm)	0.10 × 0.07 × 0.05

Data collection	
Diffractometer	Nonius KappaCCD
Absorption correction	Multi-scan (*SADABS*; Sheldrick, 2001 ▶)
*T*_min_, *T*_max_	0.942, 0.971
No. of measured, independent and observed [*I* > 2σ(*I*)] reflections	20841, 9645, 6116
*R*_int_	0.135
(sin θ/λ)_max_ (Å^−1^)	0.617

Refinement	
*R*[*F* ^2^ > 2σ(*F* ^2^)], *wR*(*F* ^2^), *S*	0.091, 0.221, 1.10
No. of reflections	9645
No. of parameters	608
No. of restraints	24
H-atom treatment	H-atom parameters constrained
Δρ_max_, Δρ_min_ (e Å^−3^)	2.94, −1.34

Computer programs: *COLLECT* (Nonius, 1998 ▶), *DENZO* and *SCALEPACK* (Otwinowski & Minor, 1997 ▶), *SORTAV* (Blessing, 1995 ▶), *SHELXS97* and *SHELXL97* (Sheldrick, 2008 ▶), *ORTEP-3 for Windows* (Farrugia, 2012 ▶) and *ATOMS* (Dowty, 1998 ▶).

## supplementary crystallographic information

### Crystal data

**Table:**

[Cd(C_15_H_18_N_2_)_2_(H_2_O)_2_](ClO_4_)_2_·C_15_H_18_N_2_·C_2_H_6_O	*Z* = 2
*M**_r_* = 1072.34	*F*(000) = 1116
Triclinic, *P*1	*D*_x_ = 1.410 Mg m^−^^3^
Hall symbol: -P 1	Mo *K*α radiation, λ = 0.71073 Å
*a* = 10.0618 (3) Å	Cell parameters from 8655 reflections
*b* = 10.1653 (3) Å	θ = 2.9–26.0°
*c* = 27.0304 (11) Å	µ = 0.60 mm^−^^1^
α = 87.163 (1)°	*T* = 120 K
β = 85.001 (1)°	Chip, colourless
γ = 66.509 (1)°	0.10 × 0.07 × 0.05 mm
*V* = 2525.60 (15) Å^3^	

### Data collection

**Table:**

Nonius KappaCCD diffractometer	9645 independent reflections
Radiation source: fine-focus sealed tube	6116 reflections with *I* > 2σ(*I*)
Graphite monochromator	*R*_int_ = 0.135
ω scans	θ_max_ = 26.0°, θ_min_ = 2.3°
Absorption correction: multi-scan (*SADABS*; Sheldrick, 2001)	*h* = −11→12
*T*_min_ = 0.942, *T*_max_ = 0.971	*k* = −12→12
20841 measured reflections	*l* = −33→32

### Refinement

**Table:**

Refinement on *F*^2^	Primary atom site location: structure-invariant direct methods
Least-squares matrix: full	Secondary atom site location: difference Fourier map
*R*[*F*^2^ > 2σ(*F*^2^)] = 0.091	Hydrogen site location: inferred from neighbouring sites
*wR*(*F*^2^) = 0.221	H-atom parameters constrained
*S* = 1.10	*w* = 1/[σ^2^(*F*_o_^2^) + (0.0516*P*)^2^ + 20.691*P*] where *P* = (*F*_o_^2^ + 2*F*_c_^2^)/3
9645 reflections	(Δ/σ)_max_ < 0.001
608 parameters	Δρ_max_ = 2.94 e Å^−^^3^
24 restraints	Δρ_min_ = −1.34 e Å^−^^3^

### Special details

**Table:**

Geometry. All e.s.d.'s (except the e.s.d. in the dihedral angle between two l.s. planes) are estimated using the full covariance matrix. The cell e.s.d.'s are taken into account individually in the estimation of e.s.d.'s in distances, angles and torsion angles; correlations between e.s.d.'s in cell parameters are only used when they are defined by crystal symmetry. An approximate (isotropic) treatment of cell e.s.d.'s is used for estimating e.s.d.'s involving l.s. planes.
Refinement. Refinement of *F*^2^ against ALL reflections. The weighted *R*-factor *wR* and goodness of fit *S* are based on *F*^2^, conventional *R*-factors *R* are based on *F*, with *F* set to zero for negative *F*^2^. The threshold expression of *F*^2^ > σ(*F*^2^) is used only for calculating *R*-factors(gt) *etc*. and is not relevant to the choice of reflections for refinement. *R*-factors based on *F*^2^ are statistically about twice as large as those based on *F*, and *R*- factors based on ALL data will be even larger.

### Fractional atomic coordinates and isotropic or equivalent isotropic displacement parameters (Å^2^)

**Table:**

	*x*	*y*	*z*	*U*_iso_*/*U*_eq_	
Cd1	0.5000	0.0000	0.0000	0.0179 (2)	
Cd2	0.0000	0.5000	0.5000	0.0152 (2)	
O1	0.3460 (6)	−0.1203 (6)	0.0086 (2)	0.0255 (14)	
H1O	0.3454	−0.1900	0.0278	0.031*	
H2O	0.2884	−0.1118	−0.0131	0.031*	
O2	−0.1216 (6)	0.3462 (6)	0.5004 (2)	0.0188 (13)	
H3O	−0.1082	0.3017	0.4730	0.023*	
H4O	−0.2117	0.3834	0.5072	0.023*	
N11	0.3004 (7)	0.2155 (7)	0.0113 (2)	0.0159 (14)	
N12	0.0329 (7)	0.4706 (7)	0.4130 (2)	0.0168 (15)	
C101	0.1756 (9)	0.2138 (9)	0.0360 (3)	0.0188 (18)	
H101	0.1650	0.1248	0.0382	0.023*	
C102	0.0667 (9)	0.3284 (9)	0.0576 (3)	0.0218 (19)	
H102	−0.0160	0.3170	0.0736	0.026*	
C103	0.0737 (9)	0.4624 (9)	0.0566 (3)	0.0180 (18)	
C104	0.1981 (9)	0.4690 (9)	0.0313 (3)	0.0215 (19)	
H104	0.2094	0.5576	0.0286	0.026*	
C105	0.3048 (9)	0.3478 (9)	0.0101 (3)	0.0227 (19)	
H105	0.3877	0.3574	−0.0065	0.027*	
C106	−0.0347 (9)	0.5862 (9)	0.0839 (3)	0.0221 (19)	
H10A	−0.1276	0.5737	0.0889	0.027*	
H10B	−0.0518	0.6745	0.0636	0.027*	
C107	0.0128 (9)	0.6049 (9)	0.1346 (3)	0.0204 (19)	
H10C	0.1061	0.6168	0.1298	0.024*	
H10D	−0.0607	0.6929	0.1502	0.024*	
C108	0.0311 (10)	0.4762 (9)	0.1694 (3)	0.025 (2)	
H10E	0.1180	0.3924	0.1574	0.030*	
H10F	−0.0545	0.4516	0.1686	0.030*	
C109	0.0473 (10)	0.5069 (9)	0.2231 (3)	0.023 (2)	
H10G	0.1307	0.5349	0.2237	0.027*	
H10H	−0.0413	0.5883	0.2356	0.027*	
C110	0.0710 (10)	0.3759 (9)	0.2574 (3)	0.027 (2)	
H11A	−0.0020	0.3368	0.2517	0.033*	
H11B	0.1684	0.3007	0.2485	0.033*	
C111	0.0598 (10)	0.4099 (9)	0.3123 (3)	0.022 (2)	
C112	0.1818 (10)	0.3809 (9)	0.3390 (3)	0.0231 (19)	
H112	0.2769	0.3403	0.3231	0.028*	
C113	0.1614 (9)	0.4124 (8)	0.3887 (3)	0.0179 (18)	
H113	0.2450	0.3909	0.4066	0.022*	
C114	−0.0851 (9)	0.5015 (9)	0.3870 (3)	0.0203 (19)	
H114	−0.1787	0.5442	0.4040	0.024*	
C115	−0.0759 (10)	0.4739 (9)	0.3371 (3)	0.0212 (19)	
H115	−0.1615	0.4986	0.3201	0.025*	
N21	0.5191 (8)	−0.0094 (8)	0.0862 (2)	0.0192 (16)	
N22	0.7871 (7)	−0.3004 (6)	0.4863 (2)	0.0113 (13)	
C201	0.5874 (9)	−0.1351 (9)	0.1092 (3)	0.0226 (19)	
H201	0.6121	−0.2209	0.0914	0.027*	
C202	0.6240 (10)	−0.1453 (10)	0.1584 (3)	0.027 (2)	
H202	0.6703	−0.2368	0.1736	0.032*	
C203	0.5927 (10)	−0.0224 (10)	0.1847 (3)	0.025 (2)	
C204	0.5167 (10)	0.1083 (10)	0.1611 (3)	0.027 (2)	
H204	0.4871	0.1955	0.1784	0.032*	
C205	0.4851 (10)	0.1102 (10)	0.1130 (3)	0.028 (2)	
H205	0.4364	0.2006	0.0974	0.034*	
C206	0.6337 (10)	−0.0278 (11)	0.2377 (3)	0.030 (2)	
H20A	0.7225	−0.1151	0.2425	0.036*	
H20B	0.6561	0.0565	0.2432	0.036*	
C207	0.5147 (10)	−0.0287 (11)	0.2752 (3)	0.027 (2)	
H20C	0.5032	−0.1204	0.2728	0.033*	
H20D	0.4224	0.0498	0.2667	0.033*	
C208	0.5406 (9)	−0.0109 (10)	0.3284 (3)	0.023 (2)	
H20E	0.5551	0.0793	0.3307	0.027*	
H20F	0.6309	−0.0912	0.3374	0.027*	
C209	0.4158 (9)	−0.0072 (9)	0.3655 (3)	0.0196 (19)	
H20G	0.4124	−0.1034	0.3675	0.024*	
H20H	0.3230	0.0614	0.3533	0.024*	
C210	0.4299 (9)	0.0364 (9)	0.4179 (3)	0.0210 (19)	
H21A	0.4452	0.1270	0.4156	0.025*	
H21B	0.3382	0.0543	0.4385	0.025*	
C211	0.5540 (9)	−0.0769 (9)	0.4432 (3)	0.0165 (18)	
C212	0.5365 (9)	−0.1941 (9)	0.4666 (3)	0.0198 (18)	
H212	0.4445	−0.2005	0.4685	0.024*	
C213	0.6524 (9)	−0.3010 (9)	0.4870 (3)	0.0172 (18)	
H213	0.6373	−0.3803	0.5026	0.021*	
C214	0.8012 (9)	−0.1840 (9)	0.4630 (3)	0.0188 (18)	
H214	0.8939	−0.1793	0.4608	0.023*	
C215	0.6890 (9)	−0.0734 (9)	0.4424 (3)	0.0195 (18)	
H215	0.7050	0.0062	0.4276	0.023*	
N31	−0.0493 (8)	1.1537 (9)	0.4252 (3)	0.0320 (19)	
N32	−0.1723 (9)	1.0904 (9)	0.0658 (3)	0.035 (2)	
C301	−0.1172 (10)	1.1686 (10)	0.3835 (4)	0.030 (2)	
H301	−0.1875	1.2604	0.3752	0.036*	
C302	−0.0885 (10)	1.0542 (10)	0.3516 (3)	0.026 (2)	
H302	−0.1425	1.0686	0.3232	0.031*	
C303	0.0188 (9)	0.9194 (9)	0.3613 (3)	0.0154 (17)	
C304	0.0963 (9)	0.9043 (9)	0.4046 (3)	0.0202 (19)	
H304	0.1717	0.8157	0.4129	0.024*	
C305	0.0566 (10)	1.0247 (10)	0.4341 (3)	0.027 (2)	
H305	0.1086	1.0150	0.4627	0.032*	
C306	0.0541 (10)	0.7921 (9)	0.3294 (3)	0.028 (2)	
H30A	−0.0241	0.8133	0.3066	0.033*	
H30B	0.0564	0.7095	0.3508	0.033*	
C307	0.1996 (10)	0.7500 (9)	0.2985 (3)	0.028 (2)	
H30C	0.2780	0.7279	0.3212	0.033*	
H30D	0.2185	0.6617	0.2802	0.033*	
C308	0.2050 (10)	0.8652 (11)	0.2619 (3)	0.033 (2)	
H30E	0.3044	0.8338	0.2455	0.040*	
H30F	0.1855	0.9536	0.2803	0.040*	
C309	0.0965 (10)	0.9005 (10)	0.2218 (3)	0.028 (2)	
H30G	−0.0037	0.9424	0.2376	0.034*	
H30H	0.1091	0.8110	0.2053	0.034*	
C310	0.1180 (11)	1.0081 (10)	0.1822 (3)	0.032 (2)	
H31A	0.1001	1.0996	0.1983	0.038*	
H31B	0.2196	0.9686	0.1676	0.038*	
C311	0.0166 (9)	1.0358 (9)	0.1418 (3)	0.0210 (19)	
C312	−0.1188 (10)	1.1552 (10)	0.1427 (4)	0.030 (2)	
H312	−0.1468	1.2201	0.1694	0.037*	
C313	−0.2088 (11)	1.1779 (10)	0.1058 (4)	0.038 (3)	
H313	−0.3002	1.2572	0.1078	0.046*	
C314	−0.0463 (10)	0.9765 (9)	0.0655 (3)	0.025 (2)	
H314	−0.0214	0.9120	0.0387	0.030*	
C315	0.0528 (10)	0.9460 (9)	0.1029 (3)	0.023 (2)	
H315	0.1424	0.8646	0.1007	0.028*	
Cl1	0.5093 (2)	0.4849 (2)	0.40637 (8)	0.0265 (5)	
O3	0.3649 (6)	0.5756 (7)	0.4264 (2)	0.0309 (15)	
O4	0.5991 (7)	0.4198 (8)	0.4469 (3)	0.0458 (19)	
O5	0.5694 (8)	0.5707 (8)	0.3756 (2)	0.0425 (19)	
O6	0.4995 (7)	0.3747 (7)	0.3767 (2)	0.0373 (17)	
Cl2	0.4508 (3)	0.5691 (2)	0.11401 (9)	0.0307 (6)	
O7	0.4223 (8)	0.4435 (7)	0.1242 (3)	0.0416 (18)	
O8	0.3993 (8)	0.6319 (8)	0.0671 (3)	0.054 (2)	
O9	0.3709 (9)	0.6750 (9)	0.1517 (3)	0.066 (3)	
O10	0.6020 (7)	0.5384 (7)	0.1153 (3)	0.0359 (16)	
C1	0.6037 (12)	0.5405 (12)	0.2465 (4)	0.047 (3)	
H1A	0.5424	0.6134	0.2239	0.071*	
H1B	0.7062	0.5157	0.2356	0.071*	
H1C	0.5840	0.5783	0.2802	0.071*	
C2	0.5708 (14)	0.4094 (12)	0.2460 (4)	0.052 (3)	
H2A	0.4658	0.4369	0.2550	0.062*	
H2B	0.5916	0.3719	0.2119	0.062*	
O11	0.6511 (11)	0.2977 (9)	0.2790 (3)	0.080 (3)	
H11	0.6325	0.3277	0.3082	0.120*	

### Atomic displacement parameters (Å^2^)

**Table:**

	*U*^11^	*U*^22^	*U*^33^	*U*^12^	*U*^13^	*U*^23^
Cd1	0.0242 (5)	0.0207 (5)	0.0097 (4)	−0.0101 (4)	−0.0019 (4)	0.0028 (4)
Cd2	0.0155 (5)	0.0196 (5)	0.0088 (4)	−0.0055 (4)	0.0000 (3)	0.0000 (3)
O1	0.031 (4)	0.030 (4)	0.024 (3)	−0.020 (3)	−0.007 (3)	0.003 (3)
O2	0.018 (3)	0.020 (3)	0.018 (3)	−0.008 (3)	0.002 (2)	−0.001 (2)
N11	0.017 (2)	0.017 (2)	0.014 (2)	−0.0080 (16)	−0.0034 (16)	0.0051 (16)
N12	0.020 (4)	0.020 (4)	0.014 (3)	−0.011 (3)	−0.001 (3)	0.000 (3)
C101	0.031 (5)	0.018 (4)	0.012 (4)	−0.015 (4)	0.000 (4)	−0.002 (3)
C102	0.023 (5)	0.031 (5)	0.012 (4)	−0.012 (4)	0.003 (3)	0.000 (4)
C103	0.025 (5)	0.022 (5)	0.006 (4)	−0.007 (4)	−0.007 (3)	0.001 (3)
C104	0.026 (5)	0.021 (5)	0.019 (4)	−0.011 (4)	0.003 (4)	−0.002 (4)
C105	0.025 (5)	0.031 (5)	0.015 (4)	−0.015 (4)	0.002 (4)	0.003 (4)
C106	0.024 (5)	0.022 (5)	0.013 (4)	−0.001 (4)	−0.001 (3)	0.001 (4)
C107	0.025 (5)	0.013 (4)	0.014 (4)	0.001 (4)	0.001 (3)	−0.003 (3)
C108	0.032 (5)	0.024 (5)	0.017 (5)	−0.010 (4)	0.000 (4)	0.001 (4)
C109	0.032 (5)	0.021 (5)	0.011 (4)	−0.008 (4)	0.005 (4)	−0.003 (3)
C110	0.039 (6)	0.027 (5)	0.008 (4)	−0.004 (4)	−0.002 (4)	0.000 (4)
C111	0.036 (5)	0.019 (4)	0.006 (4)	−0.006 (4)	0.004 (4)	0.000 (3)
C112	0.026 (5)	0.024 (5)	0.017 (4)	−0.008 (4)	−0.001 (4)	−0.002 (4)
C113	0.017 (4)	0.016 (4)	0.016 (4)	−0.001 (4)	−0.004 (3)	0.001 (3)
C114	0.021 (5)	0.028 (5)	0.018 (4)	−0.016 (4)	−0.003 (4)	−0.001 (4)
C115	0.026 (5)	0.024 (5)	0.014 (4)	−0.010 (4)	−0.006 (4)	0.001 (4)
N21	0.023 (4)	0.029 (4)	0.008 (3)	−0.014 (3)	0.002 (3)	0.003 (3)
N22	0.012 (2)	0.012 (2)	0.009 (2)	−0.0042 (16)	0.0046 (16)	−0.0034 (16)
C201	0.028 (5)	0.026 (5)	0.010 (4)	−0.007 (4)	0.000 (3)	−0.001 (4)
C202	0.029 (5)	0.031 (5)	0.015 (4)	−0.008 (4)	0.001 (4)	−0.001 (4)
C203	0.029 (5)	0.036 (6)	0.015 (4)	−0.019 (4)	0.002 (4)	−0.004 (4)
C204	0.045 (6)	0.019 (5)	0.022 (5)	−0.016 (4)	−0.006 (4)	−0.001 (4)
C205	0.042 (6)	0.025 (5)	0.022 (5)	−0.018 (5)	−0.007 (4)	0.002 (4)
C206	0.033 (6)	0.045 (6)	0.016 (5)	−0.018 (5)	−0.006 (4)	0.000 (4)
C207	0.022 (5)	0.045 (6)	0.015 (4)	−0.014 (5)	−0.002 (4)	0.000 (4)
C208	0.023 (5)	0.035 (5)	0.008 (4)	−0.009 (4)	0.000 (3)	0.003 (4)
C209	0.023 (5)	0.020 (5)	0.013 (4)	−0.007 (4)	−0.005 (3)	0.009 (3)
C210	0.021 (5)	0.025 (5)	0.012 (4)	−0.004 (4)	0.000 (3)	0.000 (4)
C211	0.019 (5)	0.025 (5)	0.004 (4)	−0.006 (4)	−0.001 (3)	−0.004 (3)
C212	0.013 (4)	0.025 (5)	0.017 (4)	−0.003 (4)	0.001 (3)	−0.004 (4)
C213	0.020 (5)	0.017 (4)	0.013 (4)	−0.006 (4)	0.004 (3)	−0.001 (3)
C214	0.018 (4)	0.023 (5)	0.015 (4)	−0.008 (4)	0.001 (3)	0.000 (3)
C215	0.026 (5)	0.021 (5)	0.010 (4)	−0.010 (4)	0.003 (3)	0.002 (3)
N31	0.026 (5)	0.038 (5)	0.034 (5)	−0.015 (4)	0.002 (4)	−0.008 (4)
N32	0.033 (5)	0.042 (5)	0.031 (5)	−0.018 (4)	−0.005 (4)	0.010 (4)
C301	0.032 (6)	0.025 (5)	0.040 (6)	−0.019 (4)	0.003 (4)	−0.005 (4)
C302	0.026 (5)	0.031 (5)	0.019 (5)	−0.008 (4)	−0.006 (4)	0.005 (4)
C303	0.019 (4)	0.025 (5)	0.007 (4)	−0.013 (4)	−0.005 (3)	0.002 (3)
C304	0.021 (4)	0.024 (4)	0.016 (4)	−0.010 (3)	−0.007 (3)	0.010 (3)
C305	0.028 (5)	0.045 (6)	0.013 (4)	−0.020 (5)	0.002 (4)	−0.008 (4)
C306	0.040 (6)	0.019 (5)	0.028 (5)	−0.016 (4)	−0.007 (4)	0.007 (4)
C307	0.026 (5)	0.024 (5)	0.030 (5)	−0.005 (4)	−0.007 (4)	−0.007 (4)
C308	0.025 (5)	0.039 (6)	0.032 (5)	−0.007 (5)	−0.006 (4)	−0.012 (5)
C309	0.032 (5)	0.035 (5)	0.016 (4)	−0.012 (4)	−0.001 (4)	−0.003 (4)
C310	0.040 (6)	0.032 (5)	0.030 (5)	−0.020 (5)	−0.007 (4)	0.003 (4)
C311	0.021 (5)	0.023 (5)	0.024 (5)	−0.016 (4)	0.007 (4)	0.002 (4)
C312	0.031 (6)	0.022 (5)	0.042 (6)	−0.014 (4)	−0.006 (5)	−0.002 (4)
C313	0.028 (6)	0.024 (5)	0.057 (7)	−0.007 (4)	0.010 (5)	0.012 (5)
C314	0.043 (6)	0.020 (5)	0.021 (5)	−0.021 (5)	−0.007 (4)	−0.001 (4)
C315	0.024 (4)	0.018 (4)	0.028 (4)	−0.009 (3)	−0.005 (3)	0.012 (3)
Cl1	0.0150 (11)	0.0345 (13)	0.0266 (12)	−0.0066 (10)	0.0037 (9)	−0.0067 (10)
O3	0.016 (3)	0.035 (4)	0.031 (4)	−0.003 (3)	0.013 (3)	−0.003 (3)
O4	0.029 (4)	0.047 (5)	0.044 (4)	0.005 (3)	−0.012 (3)	−0.002 (4)
O5	0.048 (5)	0.066 (5)	0.029 (4)	−0.041 (4)	0.015 (3)	−0.010 (3)
O6	0.028 (4)	0.042 (4)	0.043 (4)	−0.013 (3)	−0.003 (3)	−0.023 (3)
Cl2	0.0286 (13)	0.0232 (12)	0.0387 (14)	−0.0086 (10)	−0.0094 (10)	0.0107 (10)
O7	0.049 (5)	0.028 (4)	0.055 (5)	−0.024 (3)	−0.012 (4)	0.023 (3)
O8	0.055 (5)	0.051 (5)	0.061 (5)	−0.026 (4)	−0.024 (4)	0.043 (4)
O9	0.046 (5)	0.067 (6)	0.070 (6)	−0.005 (4)	0.005 (4)	−0.036 (5)
O10	0.024 (4)	0.028 (4)	0.056 (5)	−0.010 (3)	−0.010 (3)	0.000 (3)
C1	0.046 (7)	0.061 (8)	0.028 (6)	−0.016 (6)	−0.001 (5)	−0.001 (5)
C2	0.064 (8)	0.045 (7)	0.029 (6)	−0.003 (6)	−0.005 (5)	−0.003 (5)
O11	0.117 (8)	0.044 (5)	0.043 (5)	0.005 (5)	−0.003 (5)	0.001 (4)

### Geometric parameters (Å, º)

**Table:**

Cd1—O1	2.317 (5)	C207—H20D	0.9900
Cd1—O1^i^	2.317 (5)	C208—C209	1.527 (11)
Cd1—N11^i^	2.319 (7)	C208—H20E	0.9900
Cd1—N11	2.319 (7)	C208—H20F	0.9900
Cd1—N21^i^	2.349 (6)	C209—C210	1.541 (11)
Cd1—N21	2.349 (6)	C209—H20G	0.9900
Cd2—O2^ii^	2.337 (5)	C209—H20H	0.9900
Cd2—O2	2.337 (5)	C210—C211	1.508 (11)
Cd2—N22^iii^	2.333 (6)	C210—H21A	0.9900
Cd2—N22^iv^	2.333 (6)	C210—H21B	0.9900
Cd2—N12^ii^	2.363 (6)	C211—C215	1.372 (11)
Cd2—N12	2.363 (6)	C211—C212	1.387 (11)
O1—H1O	0.8595	C212—C213	1.374 (11)
O1—H2O	0.8386	C212—H212	0.9500
O2—H3O	0.8596	C213—H213	0.9500
O2—H4O	0.8387	C214—C215	1.371 (11)
N11—C105	1.362 (10)	C214—H214	0.9500
N11—C101	1.376 (10)	C215—H215	0.9500
N12—C113	1.317 (10)	N31—C301	1.340 (12)
N12—C114	1.352 (10)	N31—C305	1.347 (12)
C101—C102	1.355 (12)	N32—C314	1.333 (12)
C101—H101	0.9500	N32—C313	1.364 (13)
C102—C103	1.391 (12)	C301—C302	1.403 (13)
C102—H102	0.9500	C301—H301	0.9500
C103—C104	1.398 (11)	C302—C303	1.395 (12)
C103—C106	1.474 (11)	C302—H302	0.9500
C104—C105	1.380 (12)	C303—C304	1.431 (11)
C104—H104	0.9500	C303—C306	1.495 (12)
C105—H105	0.9500	C304—C305	1.395 (12)
C106—C107	1.535 (11)	C304—H304	0.9500
C106—H10A	0.9900	C305—H305	0.9500
C106—H10B	0.9900	C306—C307	1.531 (12)
C107—C108	1.531 (11)	C306—H30A	0.9900
C107—H10C	0.9900	C306—H30B	0.9900
C107—H10D	0.9900	C307—C308	1.510 (13)
C108—C109	1.535 (11)	C307—H30C	0.9900
C108—H10E	0.9900	C307—H30D	0.9900
C108—H10F	0.9900	C308—C309	1.532 (12)
C109—C110	1.533 (11)	C308—H30E	0.9900
C109—H10G	0.9900	C308—H30F	0.9900
C109—H10H	0.9900	C309—C310	1.556 (12)
C110—C111	1.525 (11)	C309—H30G	0.9900
C110—H11A	0.9900	C309—H30H	0.9900
C110—H11B	0.9900	C310—C311	1.498 (12)
C111—C115	1.384 (12)	C310—H31A	0.9900
C111—C112	1.400 (12)	C310—H31B	0.9900
C112—C113	1.376 (11)	C311—C315	1.355 (12)
C112—H112	0.9500	C311—C312	1.418 (13)
C113—H113	0.9500	C312—C313	1.355 (14)
C114—C115	1.379 (11)	C312—H312	0.9500
C114—H114	0.9500	C313—H313	0.9500
C115—H115	0.9500	C314—C315	1.417 (12)
N21—C201	1.337 (11)	C314—H314	0.9500
N21—C205	1.353 (11)	C315—H315	0.9500
N22—C213	1.356 (10)	Cl1—O5	1.441 (7)
N22—C214	1.365 (10)	Cl1—O4	1.443 (7)
N22—Cd2^v^	2.333 (6)	Cl1—O6	1.449 (6)
C201—C202	1.397 (11)	Cl1—O3	1.449 (6)
C201—H201	0.9500	Cl2—O7	1.425 (6)
C202—C203	1.380 (12)	Cl2—O10	1.429 (7)
C202—H202	0.9500	Cl2—O8	1.433 (7)
C203—C204	1.396 (12)	Cl2—O9	1.456 (8)
C203—C206	1.516 (11)	C1—C2	1.497 (15)
C204—C205	1.361 (12)	C1—H1A	0.9800
C204—H204	0.9500	C1—H1B	0.9800
C205—H205	0.9500	C1—H1C	0.9800
C206—C207	1.504 (12)	C2—O11	1.424 (13)
C206—H20A	0.9900	C2—H2A	0.9900
C206—H20B	0.9900	C2—H2B	0.9900
C207—C208	1.513 (11)	O11—H11	0.8400
C207—H20C	0.9900		
			
O1—Cd1—O1^i^	180.0	H20A—C206—H20B	107.9
O1—Cd1—N11^i^	90.6 (2)	C206—C207—C208	114.4 (7)
O1^i^—Cd1—N11^i^	89.4 (2)	C206—C207—H20C	108.7
O1—Cd1—N11	89.4 (2)	C208—C207—H20C	108.7
O1^i^—Cd1—N11	90.6 (2)	C206—C207—H20D	108.7
N11^i^—Cd1—N11	180.0	C208—C207—H20D	108.7
O1—Cd1—N21^i^	88.7 (2)	H20C—C207—H20D	107.6
O1^i^—Cd1—N21^i^	91.3 (2)	C207—C208—C209	113.3 (7)
N11^i^—Cd1—N21^i^	87.8 (2)	C207—C208—H20E	108.9
N11—Cd1—N21^i^	92.2 (2)	C209—C208—H20E	108.9
O1—Cd1—N21	91.3 (2)	C207—C208—H20F	108.9
O1^i^—Cd1—N21	88.7 (2)	C209—C208—H20F	108.9
N11^i^—Cd1—N21	92.2 (2)	H20E—C208—H20F	107.7
N11—Cd1—N21	87.8 (2)	C208—C209—C210	113.1 (7)
N21^i^—Cd1—N21	180.0	C208—C209—H20G	109.0
N22^iii^—Cd2—N22^iv^	180.0	C210—C209—H20G	109.0
N22^iii^—Cd2—O2^ii^	88.5 (2)	C208—C209—H20H	109.0
N22^iv^—Cd2—O2^ii^	91.5 (2)	C210—C209—H20H	109.0
N22^iii^—Cd2—O2	91.5 (2)	H20G—C209—H20H	107.8
N22^iv^—Cd2—O2	88.5 (2)	C211—C210—C209	112.4 (7)
O2^ii^—Cd2—O2	180.0	C211—C210—H21A	109.1
N22^iii^—Cd2—N12^ii^	92.9 (2)	C209—C210—H21A	109.1
N22^iv^—Cd2—N12^ii^	87.1 (2)	C211—C210—H21B	109.1
O2^ii^—Cd2—N12^ii^	85.8 (2)	C209—C210—H21B	109.1
O2—Cd2—N12^ii^	94.2 (2)	H21A—C210—H21B	107.9
N22^iii^—Cd2—N12	87.1 (2)	C215—C211—C212	117.0 (7)
N22^iv^—Cd2—N12	92.9 (2)	C215—C211—C210	122.6 (7)
O2^ii^—Cd2—N12	94.2 (2)	C212—C211—C210	120.3 (7)
O2—Cd2—N12	85.8 (2)	C213—C212—C211	119.9 (8)
N12^ii^—Cd2—N12	180.0	C213—C212—H212	120.0
Cd1—O1—H1O	131.8	C211—C212—H212	120.0
Cd1—O1—H2O	120.6	N22—C213—C212	124.0 (7)
H1O—O1—H2O	106.4	N22—C213—H213	118.0
Cd2—O2—H3O	113.7	C212—C213—H213	118.0
Cd2—O2—H4O	116.2	N22—C214—C215	123.7 (8)
H3O—O2—H4O	106.4	N22—C214—H214	118.1
C105—N11—C101	112.7 (7)	C215—C214—H214	118.1
C105—N11—Cd1	125.5 (5)	C214—C215—C211	120.5 (8)
C101—N11—Cd1	118.6 (5)	C214—C215—H215	119.8
C113—N12—C114	117.4 (7)	C211—C215—H215	119.8
C113—N12—Cd2	123.4 (5)	C301—N31—C305	117.0 (8)
C114—N12—Cd2	118.9 (5)	C314—N32—C313	117.1 (8)
C102—C101—N11	125.4 (7)	N31—C301—C302	122.7 (9)
C102—C101—H101	117.3	N31—C301—H301	118.7
N11—C101—H101	117.3	C302—C301—H301	118.7
C101—C102—C103	121.2 (8)	C303—C302—C301	120.4 (8)
C101—C102—H102	119.4	C303—C302—H302	119.8
C103—C102—H102	119.4	C301—C302—H302	119.8
C102—C103—C104	115.1 (8)	C302—C303—C304	117.2 (8)
C102—C103—C106	123.0 (8)	C302—C303—C306	123.4 (7)
C104—C103—C106	121.7 (8)	C304—C303—C306	119.3 (8)
C105—C104—C103	120.6 (8)	C305—C304—C303	117.4 (8)
C105—C104—H104	119.7	C305—C304—H304	121.3
C103—C104—H104	119.7	C303—C304—H304	121.3
N11—C105—C104	125.0 (8)	N31—C305—C304	125.2 (8)
N11—C105—H105	117.5	N31—C305—H305	117.4
C104—C105—H105	117.5	C304—C305—H305	117.4
C103—C106—C107	112.8 (7)	C303—C306—C307	113.5 (7)
C103—C106—H10A	109.0	C303—C306—H30A	108.9
C107—C106—H10A	109.0	C307—C306—H30A	108.9
C103—C106—H10B	109.0	C303—C306—H30B	108.9
C107—C106—H10B	109.0	C307—C306—H30B	108.9
H10A—C106—H10B	107.8	H30A—C306—H30B	107.7
C108—C107—C106	111.8 (7)	C308—C307—C306	113.5 (7)
C108—C107—H10C	109.2	C308—C307—H30C	108.9
C106—C107—H10C	109.2	C306—C307—H30C	108.9
C108—C107—H10D	109.2	C308—C307—H30D	108.9
C106—C107—H10D	109.2	C306—C307—H30D	108.9
H10C—C107—H10D	107.9	H30C—C307—H30D	107.7
C107—C108—C109	112.0 (7)	C307—C308—C309	114.0 (8)
C107—C108—H10E	109.2	C307—C308—H30E	108.8
C109—C108—H10E	109.2	C309—C308—H30E	108.8
C107—C108—H10F	109.2	C307—C308—H30F	108.8
C109—C108—H10F	109.2	C309—C308—H30F	108.8
H10E—C108—H10F	107.9	H30E—C308—H30F	107.7
C110—C109—C108	111.8 (7)	C308—C309—C310	111.5 (8)
C110—C109—H10G	109.3	C308—C309—H30G	109.3
C108—C109—H10G	109.3	C310—C309—H30G	109.3
C110—C109—H10H	109.3	C308—C309—H30H	109.3
C108—C109—H10H	109.3	C310—C309—H30H	109.3
H10G—C109—H10H	107.9	H30G—C309—H30H	108.0
C111—C110—C109	113.2 (7)	C311—C310—C309	111.0 (7)
C111—C110—H11A	108.9	C311—C310—H31A	109.4
C109—C110—H11A	108.9	C309—C310—H31A	109.4
C111—C110—H11B	108.9	C311—C310—H31B	109.4
C109—C110—H11B	108.9	C309—C310—H31B	109.4
H11A—C110—H11B	107.8	H31A—C310—H31B	108.0
C115—C111—C112	117.8 (7)	C315—C311—C312	117.7 (8)
C115—C111—C110	119.4 (8)	C315—C311—C310	120.4 (8)
C112—C111—C110	122.8 (8)	C312—C311—C310	121.9 (8)
C113—C112—C111	118.8 (8)	C313—C312—C311	120.9 (9)
C113—C112—H112	120.6	C313—C312—H312	119.6
C111—C112—H112	120.6	C311—C312—H312	119.6
N12—C113—C112	123.9 (8)	C312—C313—N32	122.0 (9)
N12—C113—H113	118.1	C312—C313—H313	119.0
C112—C113—H113	118.1	N32—C313—H313	119.0
N12—C114—C115	123.0 (8)	N32—C314—C315	123.8 (8)
N12—C114—H114	118.5	N32—C314—H314	118.1
C115—C114—H114	118.5	C315—C314—H314	118.1
C114—C115—C111	119.0 (8)	C311—C315—C314	118.5 (9)
C114—C115—H115	120.5	C311—C315—H315	120.8
C111—C115—H115	120.5	C314—C315—H315	120.8
C201—N21—C205	116.7 (7)	O5—Cl1—O4	110.3 (5)
C201—N21—Cd1	120.2 (5)	O5—Cl1—O6	109.5 (4)
C205—N21—Cd1	122.2 (6)	O4—Cl1—O6	109.9 (4)
C213—N22—C214	114.8 (7)	O5—Cl1—O3	109.3 (4)
C213—N22—Cd2^v^	125.4 (5)	O4—Cl1—O3	108.9 (4)
C214—N22—Cd2^v^	117.4 (5)	O6—Cl1—O3	109.0 (4)
N21—C201—C202	122.7 (8)	O7—Cl2—O10	111.3 (4)
N21—C201—H201	118.7	O7—Cl2—O8	110.6 (4)
C202—C201—H201	118.7	O10—Cl2—O8	110.9 (4)
C203—C202—C201	119.9 (8)	O7—Cl2—O9	108.9 (5)
C203—C202—H202	120.0	O10—Cl2—O9	108.3 (5)
C201—C202—H202	120.0	O8—Cl2—O9	106.9 (5)
C202—C203—C204	117.0 (8)	C2—C1—H1A	109.5
C202—C203—C206	121.9 (8)	C2—C1—H1B	109.5
C204—C203—C206	121.0 (8)	H1A—C1—H1B	109.5
C205—C204—C203	119.8 (8)	C2—C1—H1C	109.5
C205—C204—H204	120.1	H1A—C1—H1C	109.5
C203—C204—H204	120.1	H1B—C1—H1C	109.5
N21—C205—C204	123.8 (8)	O11—C2—C1	114.2 (10)
N21—C205—H205	118.1	O11—C2—H2A	108.7
C204—C205—H205	118.1	C1—C2—H2A	108.7
C207—C206—C203	112.4 (7)	O11—C2—H2B	108.7
C207—C206—H20A	109.1	C1—C2—H2B	108.7
C203—C206—H20A	109.1	H2A—C2—H2B	107.6
C207—C206—H20B	109.1	C2—O11—H11	109.5
C203—C206—H20B	109.1		
			
O1—Cd1—N11—C105	−178.7 (6)	N11^i^—Cd1—N21—C205	145.8 (7)
O1^i^—Cd1—N11—C105	1.3 (6)	N11—Cd1—N21—C205	−34.2 (7)
N11^i^—Cd1—N11—C105	−165 (5)	N21^i^—Cd1—N21—C205	61 (15)
N21^i^—Cd1—N11—C105	−90.1 (6)	C205—N21—C201—C202	−0.5 (12)
N21—Cd1—N11—C105	89.9 (6)	Cd1—N21—C201—C202	168.8 (6)
O1—Cd1—N11—C101	23.3 (6)	N21—C201—C202—C203	−1.6 (13)
O1^i^—Cd1—N11—C101	−156.7 (6)	C201—C202—C203—C204	3.8 (13)
N11^i^—Cd1—N11—C101	37 (5)	C201—C202—C203—C206	−178.4 (8)
N21^i^—Cd1—N11—C101	111.9 (6)	C202—C203—C204—C205	−4.1 (13)
N21—Cd1—N11—C101	−68.1 (6)	C206—C203—C204—C205	178.0 (8)
N22^iii^—Cd2—N12—C113	150.8 (6)	C201—N21—C205—C204	0.1 (13)
N22^iv^—Cd2—N12—C113	−29.2 (6)	Cd1—N21—C205—C204	−168.9 (7)
O2^ii^—Cd2—N12—C113	62.5 (6)	C203—C204—C205—N21	2.2 (14)
O2—Cd2—N12—C113	−117.5 (6)	C202—C203—C206—C207	−91.2 (11)
N12^ii^—Cd2—N12—C113	−95 (26)	C204—C203—C206—C207	86.5 (11)
N22^iii^—Cd2—N12—C114	−36.1 (6)	C203—C206—C207—C208	−171.0 (8)
N22^iv^—Cd2—N12—C114	143.9 (6)	C206—C207—C208—C209	178.1 (8)
O2^ii^—Cd2—N12—C114	−124.3 (6)	C207—C208—C209—C210	−170.2 (7)
O2—Cd2—N12—C114	55.7 (6)	C208—C209—C210—C211	−69.2 (9)
N12^ii^—Cd2—N12—C114	78 (26)	C209—C210—C211—C215	95.5 (9)
C105—N11—C101—C102	−0.1 (11)	C209—C210—C211—C212	−81.6 (9)
Cd1—N11—C101—C102	160.6 (7)	C215—C211—C212—C213	−1.0 (11)
N11—C101—C102—C103	−0.7 (13)	C210—C211—C212—C213	176.3 (7)
C101—C102—C103—C104	1.3 (12)	C214—N22—C213—C212	−0.5 (11)
C101—C102—C103—C106	−173.4 (8)	Cd2^v^—N22—C213—C212	−162.2 (6)
C102—C103—C104—C105	−1.2 (12)	C211—C212—C213—N22	0.5 (12)
C106—C103—C104—C105	173.6 (8)	C213—N22—C214—C215	1.3 (11)
C101—N11—C105—C104	0.2 (12)	Cd2^v^—N22—C214—C215	164.6 (6)
Cd1—N11—C105—C104	−158.9 (7)	N22—C214—C215—C211	−2.0 (13)
C103—C104—C105—N11	0.5 (13)	C212—C211—C215—C214	1.7 (12)
C102—C103—C106—C107	95.9 (10)	C210—C211—C215—C214	−175.5 (8)
C104—C103—C106—C107	−78.5 (10)	C305—N31—C301—C302	4.4 (13)
C103—C106—C107—C108	−62.7 (10)	N31—C301—C302—C303	−3.0 (14)
C106—C107—C108—C109	−168.2 (7)	C301—C302—C303—C304	0.1 (12)
C107—C108—C109—C110	−177.9 (8)	C301—C302—C303—C306	179.2 (8)
C108—C109—C110—C111	−169.4 (8)	C302—C303—C304—C305	1.1 (11)
C109—C110—C111—C115	79.7 (10)	C306—C303—C304—C305	−178.1 (7)
C109—C110—C111—C112	−99.4 (10)	C301—N31—C305—C304	−3.2 (13)
C115—C111—C112—C113	2.3 (12)	C303—C304—C305—N31	0.5 (13)
C110—C111—C112—C113	−178.6 (8)	C302—C303—C306—C307	107.9 (10)
C114—N12—C113—C112	−0.4 (12)	C304—C303—C306—C307	−73.1 (10)
Cd2—N12—C113—C112	172.9 (6)	C303—C306—C307—C308	−62.3 (10)
C111—C112—C113—N12	−1.0 (13)	C306—C307—C308—C309	−63.3 (10)
C113—N12—C114—C115	0.4 (12)	C307—C308—C309—C310	−174.2 (8)
Cd2—N12—C114—C115	−173.2 (6)	C308—C309—C310—C311	177.0 (8)
N12—C114—C115—C111	1.0 (13)	C309—C310—C311—C315	−84.8 (10)
C112—C111—C115—C114	−2.3 (12)	C309—C310—C311—C312	95.4 (10)
C110—C111—C115—C114	178.6 (8)	C315—C311—C312—C313	0.3 (12)
O1—Cd1—N21—C201	67.8 (6)	C310—C311—C312—C313	−179.9 (8)
O1^i^—Cd1—N21—C201	−112.2 (6)	C311—C312—C313—N32	−1.9 (14)
N11^i^—Cd1—N21—C201	−22.8 (6)	C314—N32—C313—C312	3.1 (13)
N11—Cd1—N21—C201	157.2 (6)	C313—N32—C314—C315	−2.9 (13)
N21^i^—Cd1—N21—C201	−107 (15)	C312—C311—C315—C314	0.0 (11)
O1—Cd1—N21—C205	−123.5 (7)	C310—C311—C315—C314	−179.9 (7)
O1^i^—Cd1—N21—C205	56.5 (7)	N32—C314—C315—C311	1.4 (13)

Symmetry codes: (i) −*x*+1, −*y*, −*z*; (ii) −*x*, −*y*+1, −*z*+1; (iii) *x*−1, *y*+1, *z*; (iv) −*x*+1, −*y*, −*z*+1; (v) *x*+1, *y*−1, *z*.

### Hydrogen-bond geometry (Å, º)

**Table:**

*D*—H···*A*	*D*—H	H···*A*	*D*···*A*	*D*—H···*A*
O1—H1*O*···O8^vi^	0.86	1.96	2.795 (9)	165
O1—H2*O*···N32^vii^	0.84	1.87	2.705 (10)	175
O2—H3*O*···N31^vi^	0.86	1.91	2.736 (9)	162
O2—H4*O*···O3^ii^	0.84	2.20	2.880 (8)	138
O11—H11···O6	0.84	2.14	2.910 (11)	152
C1—H1*C*···O5	0.98	2.57	3.493 (12)	157
C101—H101···O1	0.95	2.55	3.226 (10)	128
C113—H113···O6	0.95	2.56	3.257 (12)	130
C201—H201···O10^vi^	0.95	2.54	3.260 (11)	133
C205—H205···O7	0.95	2.55	3.214 (12)	127
C214—H214···O2^iv^	0.95	2.52	3.201 (11)	128
C304—H304···O3	0.95	2.47	3.420 (11)	174

Symmetry codes: (ii) −*x*, −*y*+1, −*z*+1; (iv) −*x*+1, −*y*, −*z*+1; (vi) *x*, *y*−1, *z*; (vii) −*x*, −*y*+1, −*z*.
